# Establishing the upper reference limit of Galectin-3 in healthy blood donors

**DOI:** 10.11613/BM.2017.030709

**Published:** 2017-10-15

**Authors:** Luisa Agnello, Chiara Bellia, Bruna Lo Sasso, Alessia Pivetti, Maddalena Muratore, Concetta Scazzone, Giulia Bivona, Giuseppe Lippi, Marcello Ciaccio

**Affiliations:** 1 Section of Clinical Biochemistry and Clinical Molecular Medicine, Department of Biopathology and Medical Biotechnologies, University of Palermo, Italy; 2 Unit of Trasfusional Medicine, Villa Sofia-Cervello Hospital, Palermo, Italy; 3 Section of Clinical Biochemistry, University of Verona, Italy; 4 UOC of Laboratory Medicine, AOUP “P. Giaccone”, Palermo, Italy; † The authors share last position.

**Keywords:** galectin-3, blood donors, heart failure, reference values, upper reference limit (URL)

## Abstract

**Introduction:**

Galectin-3 (Gal-3) is an independent predictor of poor outcomes and mortality in patients with heart failure (HF). Thus, it has been proposed as a reliable prognostic biomarker for HF. The definition of reference intervals is mandatory for interpreting the findings of experimental studies and encouraging the routine use of biomarkers in clinical practice. To date, no study assessed the reference intervals of Gal-3 and identified the biological variables that affect its concentration in a well-defined healthy population. The aim of this study was to determine the upper reference limit (URL) of Gal-3 in a highly reliable population of healthy subjects.

**Materials and methods:**

We recruited 714 blood donors. After measuring surrogate biomarkers to identify underlying diseases, 8 subjects were excluded. A final population of 706 individuals (385 men (54.5%); median age 39 (18-65) years) was included. The URL was calculated using the non-parametric percentile approach.

**Results:**

The 97.5th percentile URL of plasma Gal-3 in our study population (90% CI) was 26.1 (23.3–31.5) ng/mL. After stratifying subjects according to age, the URL of Gal-3 was found to be considerably higher in older (> 45 years) than in younger subjects (31.5 (26.2–51.4) vs 21.8 (21–26.1) ng/mL, respectively). No sex-related differences were found in Gal-3 plasma concentration.

**Conclusions:**

We established the URL of Gal-3 in a highly selected healthy population. Our findings indicate that age is an important determinant of Gal-3 plasma concentration, so that multiple diagnostic cut-offs should be preferably used according to the different age classes.

## Introduction

Galectin-3 (Gal-3) is a highly versatile lectin involved in several physiological and pathological processes including inflammation and fibrosis, which are pivotal events in development and progression of adverse cardiac remodelling and heart failure (HF) (*1*, *2*). Gal-3 is receiving growing attention due to its potential role in HF, from risk evaluation to diagnosis, prognosis and therapy. Ho *et al.* showed that increased Gal-3 concentrations were associated with an enhanced risk of new-onset HF in apparently healthy subjects recruited from the Framingham Heart Study (*3*). Many clinical studies also investigated the clinical value of Gal-3 for predicting HF in cardiac disorders such as atrial fibrillation and acute coronary syndrome, achieving controversial results (*4*-*6*). Strong evidence supports the role of Gal-3 as a prognostic biomarker in both acute and chronic HF (*7*-*9*). Notably, Gal-3 has also been recently included in the American College of Cardiology Foundation (ACCF) and the American Heart Association (AHA) Guidelines for Management of Heart Failure, as an emerging biomarker for additive risk stratification in HF patients (*10*). However, they do not deal with the problem of reference intervals.

Since Gal-3 holds great promises in diagnosis, prognostication and therapeutic management of HF, the availability of an accurate reference range and the knowledge of the variables that could influence its concentrations is essential to promote clinical research and for its diagnostic application. To date, only few studies have been published on the reference intervals of plasma Gal-3, and most of these were carried out on presumably healthy populations, not carefully characterized and without representative number of individuals (*11*-*13*). Thus, there is no consensus on the cut-off values for Gal-3.

Therefore, the aim of this study was to define the upper reference limit (URL) of plasma Gal-3 in a healthy population carefully selected by a screening method based on surrogate blood biomarkers. We chose to refer only to URL because lower reference limit has no clinical significance.

## Materials and methods

### Study design and subjects

This observational study originally included 714 consecutive blood donors recruited from the Unit of Transfusion Medicine of Villa Sofia-Cervello Hospital in Palermo, from April to July 2016. Health status of blood donors was assessed in two steps. First, the donors filled a questionnaire about their past and present health status and lifestyle. Then, they underwent to a physical examination including the collection of anthropometric parameters, arterial pressure, personal and family medical history. During physical examination a blood sample was collected for the determination of routine clinical chemistry and infectious disease testing. Admission to donation was determined according to local law. Particularly, in Italy donation criteria are published on Gazzetta Ufficiale Repubblica Italiana, 28 December 2015, ordinary supplement nr. 69.

Exclusion criteria for donation were cancer, autoimmune or cardiovascular diseases (*e.g.*, coronary artery disease, angina, cardiac arrhythmias, history of cerebrovascular diseases, arterial thrombosis, recurrent deep vein thrombosis, hypertension with organ damage), organic diseases of the central nervous system (CNS), transplant recipients, diagnosis of haemostatic disorders, epilepsy, anaphylaxis, drug use, chronic alcoholism, infectious diseases and any chronic hepatic, gastrointestinal, urogenital, haematologic, immunologic, renal, metabolic and respiratory disorder. Diabetics with good glycemic control and not needing insulin treatment were admitted for donation. Glycemic control was evaluated based on self-reported HbA1c < 53 mmol/L, preprandial plasma glucose between 4.4 and 7.2 mmol/L, and postprandial plasma glucose < 10 mmol/L according to American Diabetes Association Diabetes (ADA) guidelines (*14*). Selected biomarkers were used to identify clinically asymptomatic diseases, and further refine the study population. In particular, we used B-type natriuretic peptide (BNP) and high sensitivity troponin I (hsTnI) to assess myocardial dysfunction, and creatinine for estimated Glomerular Filtration Rate (eGFR) calculation to assess kidney disease. Plasma concentration of BNP > 104 pg/mL in men and > 150 pg/mL in women, plasma concentration of hsTnI > 34.2 ng/L in men and > 15.6 ng/L in women, as for manufacturer cut-offs; and an eGFR < 60 mL/min/1.73m^2^ were applied as exclusion criteria, according to The Kidney Disease: Improving Global Outcomes (KDIGO) guidelines (*15*). The study protocol was approved by the local ethics committee and was performed in accordance with the Declaration of Helsinki.

Overall 8 subjects from the original population of 714 blood donors ought to be excluded due to values of hsTnI (N = 7) or BNP (N = 1) above the respective URL. None of the subjects had an eGFR ≤ 60 mL/min/1.73m^2^. Therefore, the final study population consisted of 706 subjects, aged from 18 to 65 years, with a median age of 39 years (interquartile range, IQR: 18 – 65 years) and with a slightly higher rate of men (N = 385, *i.e.* 54.5%). All study participants gave an informed consent.

### Methods

Blood samples were collected in tube containing K_2_EDTA (Greiner Bio-One GmbH, 454086, Kremsmuenster, Austria) in the morning after night fasting (8 hours). Upon arrival at the Transfusion Medicine Unit, K_2_EDTA plasma was separated by centrifugation for 10 minutes at 3000 rpm at room temperature and subsequently frozen in aliquots at - 80°C until testing. In particular, we analyzed plasma samples within 3 months of the collection because BNP has a stability ≤ 3 months at - 20°C or colder, as stated by manufacturer; the other analytes are stable within this interval. The concentration of hsTnI was measured using the Architect STAT High Sensitive Troponin-I assay, which has a limit of detection (LoD) comprised between 1.1-1.9 ng/L and an imprecision (CV %) lower than 10% at the 99^th^ percentile of healthy subjects distribution, as declared by the manufacturer. The concentration of BNP was measured by the Architect BNP assay, which displays a LoD < 10 pg/mL and an imprecision (CV %) < 12%, as declared by manufacturer. Gal-3 was measured by the Architect STAT Galectin-3 immunoassay. This assay is characterized by a LoD of 1 ng/mL and a total CV ≤ 6.2% for Gal-3 values between 8.5 and 94.4 ng/mL, as declared by manufacturer. Measurements of hsTnI, BNP and Gal-3 were performed on Architect i1000 instrument (Abbott Laboratories, Wiesbaden, Germany). A calibration was performed before using a new lot number for each of these assays. A single sample of low, medium and high concentration control was tested to evaluate the assay calibration once every 24 hours each day of use. Serum creatinine was measured using the Architect C8000 instrument and reagents (Abbott Laboratories, Wiesbaden, Germany) based on a kinetic alkaline picrate method, traceable to isotope dilution mass spectrometry (IDMS) method and NIST SRM 967 standard. Estimated glomerular filtration rate (eGFR) was calculated using the Chronic Kidney Disease - Epidemiology Collaboration (CKD-EPI) equation (*16*). We took into account situations that could limit the use of GFR estimating equations.

The methods were sensitive to haemolysis, lipemic material, fibrin and other particular material including cryoprecipitate. In this cases the manufacturer suggests performing a particular procedure before testing sample to ensure consistency in results. However, we did not have unacceptable samples.

### Statistical analysis

Data were statistically analyzed with SPSS Software 15.0 (SPSS Inc., Chicago, IL, USA). Non-normally distributed continuous variables were reported as median and interquartile range (IQR). Categorical variables were shown as percentage. Normality of distribution was assessed using the Kolmogorov-Smirnov test. Differences between normally distributed variables were evaluated using ANOVA. Differences in Gal-3 concentrations according to age, sex and eGFR groups were evaluated by the non-parametric Kruskal - Wallis test because Gal-3 was non-normally distributed. The correlation between Gal-3 and age was evaluated by the Spearman rank correlation test. The URL was finally calculated with the non-parametric percentile method, according to the Clinical and Laboratory Standard Institute – CSLI C28-A3 document, considering values in the 97.5% of the distribution (*17*). This method is recommended for asymmetric distribution of non-normally distributed variables, such as for Galectin-3 in our study. The statistical significance of P < 0.05 was accepted for all tests.

## Results

**Figure 1:**
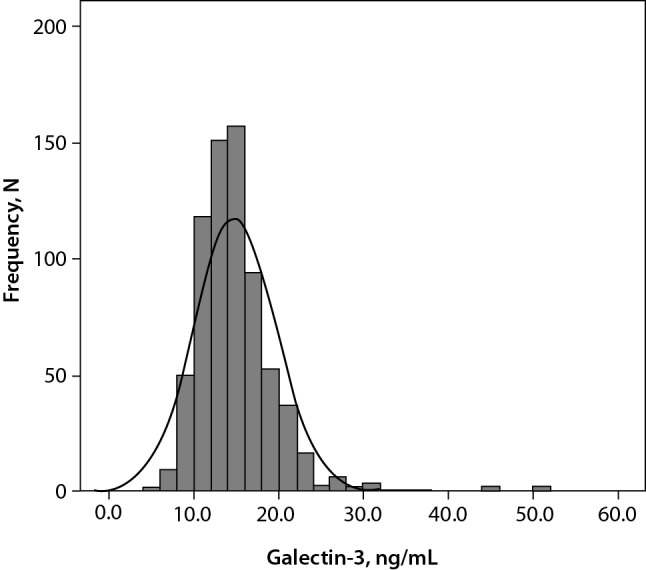
Distribution of galectin-3 concentrations

None of the subjects included in this study displayed a Gal-3 plasma concentration lower than the LoD of the assay. A non-Gaussian (right-skewed) distribution of Gal-3 values was observed (Figure 1).

**Figure 2:**
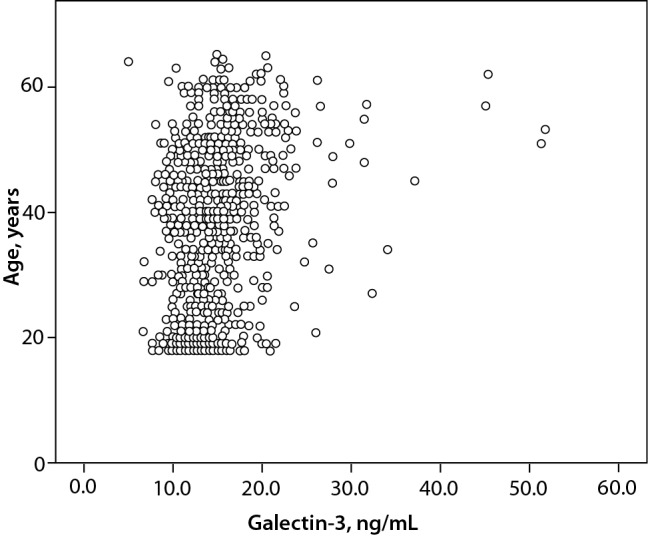
Scatter plot of the relationship between age and galectin-3 concentrations

Notably, the plasma concentration of Gal-3 was not found to be statistically different between men and women (14.4 (12.4–16.5) ng/mL *vs* 14.1 (11.5–16.9) ng/mL; P = 0.19), whereas plasma Gal-3 values were found to be significantly correlated with age (r = 0.27; P < 0.01) (Figure 2).

In this study population, the vast majority of subjects had eGFR > 90 mL/min/1.73m^2^, with only 117 subjects (16.6%) having eGFR between 60 and 89 mL/min/1.73m^2^. After stratifying the population according to eGFR values, we found that subjects with modestly decreased renal function (eGFR 60-89 mL/min/1.73m^2^) had slightly higher Gal-3 plasma values than those with normal eGFR (eGFR > 90 mL/min/1.73m^2^) (15.2 (12.5–18) ng/mL *vs* 14.1 (11.8–16.4) ng/mL; P = 0.004).

The URL of Gal-3 is shown in Table 1. When subjects were stratified according to their age, the URL of Gal-3 were considerably higher in older (*i.e.*, > 45 years) compared to younger subjects (P < 0.001). The choice of 45 years as cut-off age was arbitrary. Given the correlation between Gal-3 and age and the higher prevalence of cardiovascular disease in aged populations, we decided to identify an age specific reference limit. Therefore, the choice of this cut-off allowed us to analyze an older population without limiting the statistical significance of the analysis due to a very small sample.

**Table 1:** Upper reference limit of plasma galectin-3 concentration

**Subjects**	**N**	**Galectin-3 concentrations (ng/mL)**	
**Median (IQR)**	**97.5^th^ percentile (90% CI)**
All	706	14.3 (11.9 – 16.7)	26.1 (23.3 – 31.5)
Age ≤ 45 years	470	13.6 (11.5 – 15.9)	21.8 (21 – 26.1)
Age > 45 years	236	15.5 (12.9 – 18.3)*	31.5 (26.2 – 51.4)*
IQR - interquartile range. CI -confidence interval. *P < 0.001 when compared with subjects aged ≤ 45 years.			

## Discussion

In this study we defined the URL of Gal-3 plasma concentrations in a reference population representing “truly” healthy individuals, including blood donors strictly selected by using surrogate biomarkers. Moreover, the study population is ethnically homogeneous, only consisting of Caucasian subjects born in our country (Italy), and holding an almost identical number of men and women. The most relevant finding of our study is the age-related difference in Gal-3 plasma concentration. Additionally, we found an association between Gal-3 plasma levels and eGFR. Finally, we did not find sex-related differences in Gal-3 concentrations.

Galectin-3 is a biomarker of inflammation and fibrosis, displaying a well-established prognostic value in HF patients (*18*). The clinical use of this biomarker has been proposed along with natriuretic peptides to identify patients at higher risk of re-admission or death of HF (*10*). Nevertheless, before the measurement of Gal-3 can be thoughtfully introduced in clinical practice, it is imperative to identify its reference values and the potential influence of biological variables on its circulating levels in the general population of healthy subjects.

The URL of plasma Gal-3, namely the 97.5th percentile of the distribution, identified in our study was 26.1 ng/mL. In a previous study, Gaze *et al.* estimated a quite similar URL value (28.4 ng/mL) in 627 apparently healthy individuals (*11*). Unlike this data, Krintus *et al.* and La’ulu *et al.* calculated a much lower URL in their study populations (18.1 ng/mL and 18.7 ng/mL, respectively) (*12*, *19*). Notably, these two studies were performed using the same Galectin-3 assay (Abbott) used in our investigation. Similarly to Mueller *et al.* we found an inverse association between Gal-3 plasma values and eGFR (*20*). The relation between Gal-3 and renal function has been previously documented. Gal-3 has been linked to development of renal fibrosis in animal models, and was found to be inversely correlated with eGFR in humans (*21*, *22*). In our study, subjects with a modestly decreased renal function (eGFR 60-89 mL/min/1.73m^2^) were not excluded from the reference population, since this aspect had an insignificant impact on Gal-3 plasma concentrations.

Interestingly, we failed to find sex-related differences in plasma Gal-3 concentrations in our highly selected study population. This finding is in accordance with data published by Krintus *et al.*, but it is in disagreement with those of La’ulu *et al.* and Gaze *et al.*, who found higher Gal-3 concentrations in men, as well as with those of Mueller *et al.*, who found higher Gal-3 concentrations in women than in men (*11*, *12*, *19*, *20*). Finally, a strong correlation between Gal-3 plasma concentration and age was found in our study, with increased Gal-3 concentrations in older subjects (> 45 years). This is in accordance with previous findings (*11*, *19*, *20*). In particular, Gaze *et al.* stratified the study population by age (≥ 50 years and < 50 years), and observed a consistent trend, with increasing Gal-3 concentrations in parallel with ageing, thus finally concluding that two different URLs may be used in subjects ≥ 50 years or in those < 50 years (35.1 ng/mL and 25.5 ng/mL, respectively) (*11*). Krintus *et al.* found that the URL of Gal-3 was only slightly higher in older (≥ 40 years) compared to younger subjects (18.8 *vs* 17.9 ng/mL, respectively) (*19*). Mueller *et al.* also found a weak but still statistically significant association between Gal-3 concentrations and age (*20*).

To the best of our knowledge, our study is the first that allow defining reference values of Gal-3 in a highly reliable reference population, using strict criteria for including only healthy subjects and with a representative number of individuals in different age and sex categories.

The discrepancies observed among our findings and data of previous studies may be justified by some important aspects. First, the selection of a “really” healthy population is essential for accurately defining reference values of any clinically usable biomarker. However, how to precisely define a “healthy” reference population is currently debatable (*23*, *24*). Apple and Collinson recently defined what should constitute a healthy reference population, by emphasizing that the selection should be based on history of medications and the presence of a known underlying disease, as well as by identifying the possible presence of occult organ dysfunction by measuring some surrogate biomarkers (*25*). The vast majority of the studies aimed to define the reference values of Gal-3 lacks an accurate description of the study population, but rather simply indicate that the subjects were “healthy without known cardiac disease” (*11*-*13*). Only two studies so far have accurately described the reference “healthy” population, also clearly applying stringent exclusion criteria (*19*, *20*). However, both studies mainly included young individuals (≤ 45 years), whereas an appropriate number of older subjects was included in our investigation. This difference could hence explain the higher Gal-3 URL observed in our study population. A strong association between Gal-3 and age has been earlier reported by de Boer *et al.* in a large sample of the general population (*26*).

The sample size is another well-established critical issue for accurately defining the reference values of biomarkers. The CLSI recommends a minimum of 120 individuals (*17*). This number is mandatory to use a nonparametric estimation method for calculating the limits of a central 95% reference interval with upper limit at 97.5^th^ percentile. When stratifying the population according to possible determinants, each homogenous subclass should hence be represented by not less than 120 individuals. Therefore, when age- and sex-related differences need to be considered, the minimum number of healthy individuals escalates in parallel. Accordingly, the considerable number of subjects in each of the different population clusters (stratified by age, sex and renal function) should be seen as a major strength of our study. A potential limitation is the inclusion of only Caucasian individuals, which may hamper data transferability to other ethnic groups.

In conclusion, this is the first study aimed to define the URL of Gal-3 performed on a highly selected healthy population, stratified according to age and sex. Our findings clearly suggest that age may be an important determinant of Gal-3 plasma concentration, so that multiple diagnostic cut-offs should be preferably used according to the different age classes.
